# Postural Analysis Using Rasterstereography and Inertial Measurement Units in Volleyball Players: Different Roles as Indicators of Injury Predisposition

**DOI:** 10.3390/medicina59122102

**Published:** 2023-11-30

**Authors:** Francesco Agostini, Alessandro de Sire, Luca Furcas, Nikolaos Finamore, Giacomo Farì, Sara Giuliani, Valerio Sveva, Andrea Bernetti, Marco Paoloni, Massimiliano Mangone

**Affiliations:** 1 Department of Anatomy, Histology, Forensic Medicine and Orthopedics, Sapienza University, 00185 Rome, Italy; luca.furcas@uniroma1.it (L.F.); nikolaos.finamore@uniroma1.it (N.F.); giuliani.1840762@studenti.uniroma1.it (S.G.); valerio.sveva@uniroma1.it (V.S.); marco.paoloni@uniroma1.it (M.P.); massimiliano.mangone@uniroma1.it (M.M.); 2 Department of Neurological and Rehabilitation Science, IRCCS San Raffaele, 00166 Rome, Italy; 3 Physical Medicine and Rehabilitation Unit, Department of Medical and Surgical Sciences, University of Catanzaro “Magna Graecia”, 88100 Catanzaro, Italy; alessandro.desire@unicz.it; 4 Research Center on Musculoskeletal Health, MusculoSkeletalHealth@UMG, University of Catanzaro “Magna Graecia”, 88100 Catanzaro, Italy; 5 Department of Biological and Environmental Sciences and Technologies (DiSTeBA), University of Salento, 73100 Lecce, Italy; dr.giacomofari@gmail.com (G.F.); andrea.bernetti@unisalento.it (A.B.)

**Keywords:** volleyball players, rasterstereography, IMU, prevention, injuries, postural analysis

## Abstract

*Background and Objectives*: Acute and chronic injuries are frequent in volleyball. Biomechanics of sport-specific tasks can influence the risk of injury, which is also related to specific court positions. We investigated posture at raster-stereography, balance, and dynamic tasks using inertial motion units to find differences between roles, which can be predictive of a higher risk of injury. *Materials and Methods*: We cross-sectionally evaluated amateur volleyball athletes. Participants were divided into roles as outside hitters, setters, middle blockers, and opposite hitters. We excluded the “libero” position from our analysis. *Results*: Sixteen players were included in the analysis. A statistically significant difference was found in left lower limb stiffness among the outside hitter and setter groups. *Conclusions*: Differences in stiffness might be related to the different training and the different abilities among the two groups. Raster-stereography is extending its indications and should be implemented for non-invasive postural analysis. The use of inertial motion units provides objective measurements of variables that could go unrecognized within a clinical evaluation; its use should be considered in injury preventive programs.

## 1. Introduction

Volleyball is a popular, widespread team sport played all over the world. It is a limited-contact sport, but it requires repetitive overhead and jumping motions, which can lead to both acute and overuse injuries of the upper and lower extremities [1]. Typical actions are sprints, jumps, hits, and multidirectional movements. However, actions that allow scoring a point (spike, block, and serve) are mostly performed while jumping, and the number of jumps varies in terms of players’ roles because of different technical-tactical and motor requirements [2]. As with all sports, players are at risk of injury [3], and prevention programs are still evolving due to a lack of understanding of the specific risk factors [4].

There are no differences in injury rates among elite male and female volleyball players, as stated from a 4-year data collection from the International Volleyball Federation (FIVB) Injury Surveillance System (ISS) [5]. In another recent systematic review and meta-analysis, no gender differences were highlighted in overall injuries during volleyball matches [6].

Ankle, knee, and shoulder are the most involved districts, with slight differences in the epidemiology between indoor and outdoor games [7]. The most reported injuries are ankle sprains and acute injuries that occur most often at the net when landing after blocking or attacking [4]. However, overuse conditions have been reported as more common than acute traumas and are related to repetitive, incorrect athletic gestures [4,8]. Anterior cruciate ligament injuries, patellar tendinopathies, shoulder overuse pathologies, glenohumeral dislocation/subluxation and its correlated articular instability, wrist tendonitis, finger sprains, and dislocations enter this category [9,10]. Other types of injuries in volleyball are related to the athletic gesture during the spike and are linked to the spine, such as muscle strains of the back, intervertebral disc injuries, and spondylosis [1]. Concussion of the head is a not-so-common type of injury that could happen by crushing into the advertisement panels surrounding the court after defending a rival spike, or maybe they are associated with ball-to-head contact [11].

The risk of injury varies between players: 89% of the injuries related to a specific court position occur at the net. So, roles and positions in the court might be predictive of a different risk of injury [3].

We can recognize five different roles in volleyball:

(1) Outside hitter (spiker): placed on the left lateral side of the court, this player spikes the ball into the opposing court. He is the lead attacker of the team.
(2) Opposite hitter: this player is the right-side hitter; he plays on the opposite side than the setter and is involved both in the defense and offense phases.
(3) Setter: this player receives the ball from the passer and sets it to the hitter.
(4) Passer (libero): he wears a different T-shirt. This player receives the other team’s serve and passes the ball to the setter.
(5) Middle Blocker: this player typically plays near the net and is responsible for blocking shots from the opponents. Usually, he is the tallest player on the team.

The different risks of injury can be related to the contact (possibly while attacking or defending at the net) but also to the different movements that players perform on the court, called sport-specific skills [12]. Serving, passing, setting, and spiking require different biomechanics, and each type of player is exposed to injuries related to their roles [13]. Besides this, serving and spiking constitute a series of asymmetric movements, which impose adverse effects on the body posture [14], where a high-level player needs to perform repeatedly and for a long period in unbalanced positions, which are associated with dynamic loading [15]. Spinal twisting, anterior-posterior bending, and asymmetrical motions during these sport-specific skills are the main elements that contribute to the increase of postural instability and could be linked with sport-specific injuries.

A recent study has investigated balance and vertical jumping performance to detect differences in female athletes with different training levels (active versus retired players), highlighting that age and training level might influence balance [16]. Furthermore, in another study, athletes were assessed with a rasterstereographic analysis of the back with the aim of identifying unknown postures consequent to the reiterated repetition of specific movements [17].

Rasterstereography is an optical measurement system that provides a reliable method for three-dimensional analysis of the back and reconstruction of spinal deformities without radiation exposure. This system allows a three-dimensional reconstruction of spinal posture and pelvic position starting from the analysis of the posterior surface during orthostasis [18]. This radiation-free system provides information that correlates well, on the sagittal plane, with radiographic data and that can be used over time to perform postural analysis and evaluate the effects of therapies [19]. This technique could be used to assess the postural characteristics of athletes noninvasively [20].

Another important advancement in assessing sport-specific skills preventing injuries is the use of inertial measurement units (IMUs) composed of an accelerometer, gyroscope, and a magnetometer [21]. In a recent study, IMUs were utilized to quantify the most intense jumping movements occurring in volleyball players [22].

We hypothesized that differences between roles on sport-specific skills might play a role in static and dynamic parameters, which could influence the risk of injuries.

Therefore, the aim of this study was to investigate differences between volleyball roles on static posture with rasterstereographic analysis that are linked with differences on a static one-leg balance test and on dynamic jump skills evaluated with an IMU to find differences between roles and to determine whether these differences might expose players to a higher risk of injury.

## 2. Materials and Methods

### 2.1. Sample Size Calculation

Sample size was computed using statistical power analysis G*Power 3.1 (Heinrich Heine Universität Düsseldorf, Düsseldorf, Germany) software through a within factors test—repeated measures ANOVA for 1 group of healthy subjects (HS) with a total of 39 different dependent variables, with an α < 0.05, power (1 − β) > 0.95, correlation among repeated measures of 0.5, a non-sphericity correction ε = 1 and an hypothetical effect size f = 0.2. A total sample size of 14 subjects with an actual power of 0.965, critical F of 1.429, and non-centrality parameter λ of 43.68 with 38 degrees of freedom was calculated.

In this cross-sectional study, we included volleyball players from the minor leagues of the Italian national championship. Sixteen players from different teams of the minor Italian volleyball championship were set for inclusion and enrolled in the study. Five participants played as outside hitters, five as opposite hitters, three as setters, and three as middle blockers.

All players trained from 3 to 4 sessions per week, with each session lasting from 2 up to 3 h. All the included participants were experienced players with a mean of 9 (±3) years of practice.

We excluded the “libero” position from our evaluation since these players less commonly suffer injuries and because these players do not perform the majority of volleyball fundamentals (i.e., block, attack, and serve).

Participants were divided into 4 groups, depending on their actual team role:

(1) Outside Hitter (Outside Spiker);
(2) Setter;
(3) Middle Blocker;
(4) Opposite hitter (Opposite Spiker).

**Figure 1:**
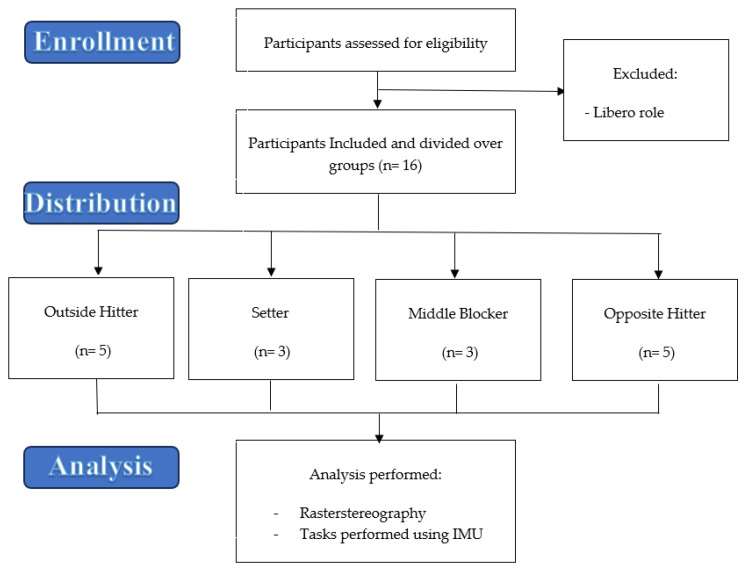
Flow chart representing the protocol for including participants.

All the included subjects took part in the following:

(1) Postural examination obtained with a rasterstereographic analysis method.
(2) Postural assessment during a static standing test and during two different dynamic jump tasks (Counter Movement Jump—CMJ and Stiffness Test—ST), both obtained using an IMU. The flow chart in “Figure 1” reports our selection protocol.

We performed our study between 1 January and 1 April 2023 at the Movement and Gait Analysis Laboratory at the Physical and Rehabilitative Medicine Department, Sapienza University, Rome, Italy.

The age, sex, height, and weight of the players were collected. Data from the participants concerning the number and type of injuries suffered during their years of practice were also collected.

### 2.2. Postural Examination with a Rasterstereographic Analysis Method

Postural alterations were evaluated with Formetric 4D System (DIERS, International GmbH, Schlangenbad, Germany), a digital spinometry that uses a non-invasive, objective, radiation-free rasterstereographic analysis.

This device projects onto the patient’s back a series of parallel light stripes that are emitted by a slide projector. A three-dimensional reconstruction of the back surface is made using triangulation equations by transforming the stripes and their corresponding curvature into a scatter plot [23]. Vertebra prominent (VP) and right (DR) and left (DL) lumbar dimples are specific back surface landmarks that are recognized automatically with a standard deviation of ±1 mm for the purposes of creating a Cartesian coordinate system, so no passive markers are necessary when using Formetric 4D [24].

Subjects were placed in a standing position, barefoot with their knees extended and their arms left naturally alongside their hips, without wearing shoes or necklaces, with the back naked. To standardize subjects’ positioning, a horizontal line was drawn on the floor in order to provide a reference for subjects’ heels (2 m away from the device).

Sample timing of a single analysis with Formetric 4D is only 40 msec [25]. To reduce errors, we acquired 12 samples for each subject in a period of 3 s (approximately the time needed for a single deep breath), averaging the mean values of all of the Formetric 4D features.

Postural features obtained with Formetric 4D were:

(1) On Frontal Plane:
  - VP-DM length (mm): distance between C7 and the mean point between right and left lumbar dimples;
  - VP-DM lateral flexion (mm): distance between the vertical line passing from C7 and the mean point between right and left lumbar dimples;
  - DL-DR pelvic inclination (mm): height gap between right and left lumbar dimples;
  - Superficial rotation of the vertebral bodies (°);
  - VP-DM lateral deviation (mm): distance between the center of the vertebral bodies and the line that passes between C7 and the mean point between right and left lumbar dimples.
(2) On Sagittal Plane:
  - Cervical and Lumbar Stagnara arrows (mm): distance between the vertical line that passes from the occipital bone to the intergluteal fold and the maximum point of cervical and lumbar lordosis;
  - Dorsal kyphotic and cervical and lumbar lordotic angles (°);
  - Pelvic tilt (°);
  - VP-DM antero-posterior flexion.

### 2.3. Postural Assessment during Static and Dynamic Tasks Using an IMU

A wireless and wearable IMU, G-SENSOR (BTS Engineering, Milan, Italy), was used to obtain data from static postural standing and during 2 different dynamic jump tasks in the group of volleyball players. G-SENSOR was built with a triaxial accelerometer, a triaxial gyroscope, and a magnetometer. Using a dedicated belt around the waist, we applied G-SENSOR in the mean position of the right and left lumbar dimples (S1–S2 vertebra).

(1) Static postural standing task: with open eyes, we obtained a postural standing recording with both right- or left-bearing legs for 60 s. Samples were rejected and repeated if the subject lost his balance or touched the ground with the other leg.

Static parameters evaluated during this task were:

- Angle between the vertical axis and principal axis of the ellipse (°);
- Total length of the trajectory of COM (mm);
- Ninety-five % confident ellipse area (mm^2^);
- Antero-posterior oscillation range of COM (mm);
- Medial-lateral oscillation range of COM (mm);
- Mean velocity of medial-lateral oscillation of COM (mm/s);
- LFS index: length of the COM divided for the area.

(2) Among the several tests that can be found in the literature, we chose the Counter Movement Jump (CMJ) test and the Stiffness Test (ST), as these tests are good examples of dynamic tasks that occur during a volleyball match or in training sessions.
(2a) Counter Movement Jump Test (CMJ) provides an assessment of explosive-elastic power in an athlete’s lower limb. A concentric contraction is done after a brief eccentric contraction phase. The subject starts the test in an orthostatic position, with their feet at shoulder-width apart and their hands on their waist. Moreover, before jumping vertically, the subject performs a countermovement bending on their knees at 90°.

Features evaluated with this jumping test were:

- Maximum jumping height (cm);
- Maximum jumping force (kN);
- Velocity peak (m/s);
- Total power (W);
- Stiffness (N/m).

(2b) Stiffness Test (ST) provides information on athletes’ muscle and tendon stiffness. The subject starts the test in an orthostatic position, with their feet shoulder-width apart and their hands on their waist. When the operator gives the start signal, the subject performs a series of vertical jumps in a prefixed time. Only the first jump can be performed with a countermovement of bending on the knee, while the other jumps have to be done with extended lower limbs. It is important that the evaluated athlete stays as much as he/she can without their feet on the ground, minimizing ground contact times.

Features evaluated with this jumping test in the right and left lower limb were:

- Velocity peak (m/s);
- Maximum jumping height (cm);
- Reactivity index;
- Impact index;
- Stiffness (N/m);
- Force (kN);
- Power (W);
- Take-off force (kN).

### 2.4. Statistical Analysis and Paper Report

All statistical analyses were performed using SPSS Statistics v.27 (IBM, Armonk, NY, USA) software. Descriptive statistics were used to describe the data collected by the sample: mean/median (standard deviation/interquartile range) for continuous variables and frequency (percentage) for dichotomous variables. The normal distribution of the collected data was checked using the Shapiro-Wilk test. Differences between the four groups in the study were analyzed with parametric and non-parametric tests, depending on the distribution of the variables: the ANOVA (Analysis of Variance) test for parametric variables and the Kruskal-Wallis test for non-parametric variables. A Tukey HSD pairwise analysis was conducted in case of significant interactions. Statistical significance was set at a = 0.05.

This study was conducted following the STROBE guidelines.

## 3. Results

The mean (standard deviation) age of the included players was 21.13 years (±1.70); the height was 170 cm (±10) for female players, 180 cm (±5) for male players; mean weight was 56.6 kg (±5) for female players, 78.5 kg (±4) for male players. All participants were right-handed.

Other demographic characteristics have been synthesized in Table 1.

### 3.1. Rasterstereographic Analysis Results

Results of the Formetric 4D analysis of the spinal posture and pelvic position have been synthesized in Table 2.

No one of the analyzed features reached a statistically significant difference between groups.

### 3.2. IMU Results

Results obtained from the IMU were separately analyzed.

The results obtained from a static postural standing task have been synthesized in Table 3. No statistically significant differences have been found between groups.

Results obtained from the CMJ task have been synthesized in Table 4. No statistically significant differences have been found between groups.

Results obtained from the Stiffness Test have been synthesized in Table 5.

**Figure 2:**
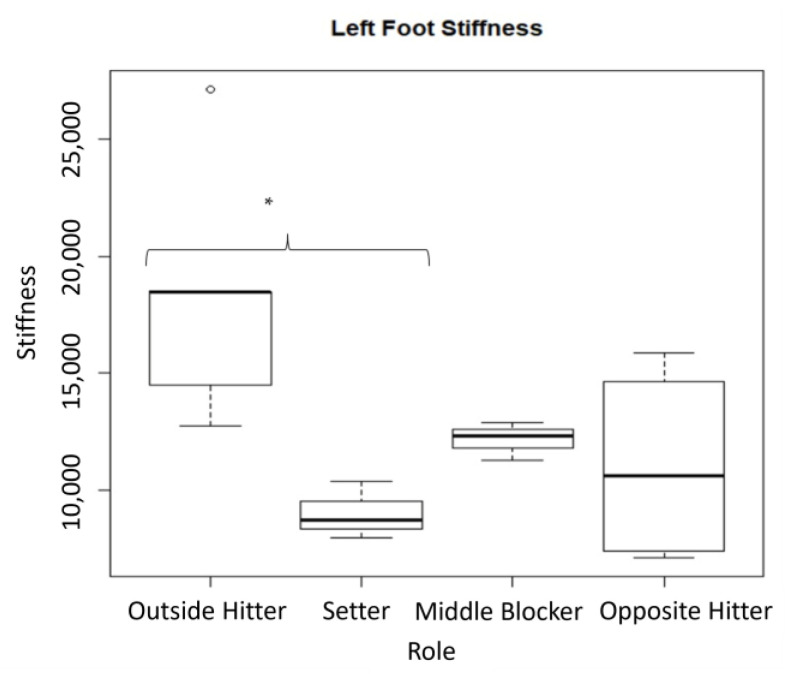
Left lower limb stiffness between groups using an IMU, *p* = 0.0287. Post-hoc analysis showed a difference between the setter group and the outside hitter group. * = statistically significant; Circle = outlier.

A statistically significant difference has been found between groups in terms of left lower limb stiffness (*p* = 0.02) (Figure 2).

The subsequent Tukey HSD post-hoc analysis showed a difference between the setter group and the outside hitter group (*p* = 0.03), with the outside hitter group showing a higher stiffness.

For an effect size evaluation, we also evaluated the mean difference between the outside hitter and setter groups, with a mean difference of −9255 (C.I.: −14,991.05–3519.2472).

## 4. Discussion

Volleyball players have to play numerous competitive matches during a year with repeated numbers of high-intensity and explosive activities, such as jumps and rapid changes in direction, interspersed with brief periods of low to moderate active recovery [26]. Despite not being as common as in contact games, injuries are very frequent in volleyball, and several preventive training and injury management strategies have been studied over the years in different ways [27]. For example, several interventional strategies have been proposed in an effort to reduce the risk of ankle sprains, working on technical training, neuromuscular and proprioceptive training, and using external ankle supports [28]. Recovery has been studied as a preventive factor [27]. Injury prevention programs might be effective in reducing some biomechanical risk factors [29] by controlling the mechanical environment of the movements required by sport-specific skills [30].

Furthermore, biomechanics can affect injuries [30]. One recent study conducted on female athletes of several sports suggested that biomechanical changes during maturation in female athletes might contribute to creating an injury risk profile [29,31]. A previous study investigated differences in physical performance among roles in volleyball players, highlighting that agility measures might show positional differences. However, they did not perform an assessment of the shape of the spine with raster-stereography. The tasks performed were different, and they did not assess the agility with the use of inertial motion units [32].

In recent years, sports science research has developed an innovative approach to different kinematic and kinetic sport gesture analyses with the aim of assessing, preventing, and even prognosticating athletic injuries [33]. In this framework, the employment of raster-stereography and IMUs in volleyball players could help coaches and trainers prevent injuries by analyzing different technical efforts in distinct roles.

In the present study, we found a statistically significant difference between roles in our group of volleyball players in the left lower limb stiffness during a dynamic test using an IMU for evaluating kinematic-associated variables. In particular, a statistical difference was encountered between the outside hitter and the setter during the ST dynamic test, and the middle blocker and opposite hitter did not reach statistical significance, but they had a higher trend compared to the setter. This result could be explained by the biomechanical and technical efforts of these two distinctive roles in volleyball. Outside hitters, as well as opposite hitters and middle-blockers, are more prone to develop ankle sprains because of their continuous risk of ankle stress during falls after serving or spiking. Instead, setters play the second touch of the game, and they have the goal of lifting up the ball for the successive spike by the hitters or by the middle-blocker. For this reason, setters have a lower prevalence of ankle sprains during the match compared to the other volleyball roles. From a biomechanical point of view, this difference could be seen in the ground reaction force (GRF) generated during the phases of a spike in an outside hitter. The non-dominant foot, before jumping, has an enormous potential force derived from the kinetic force developed during the first phase of the spike. Furthermore, the non-dominant foot is really far from the COM with an angle of 30° of internal rotation, and this increases the risk of high-inversion stress in this articulation, producing ankle instability [34].

Moreover, after an ankle sprain, recovery is not always the same and could lead to different kinds of rigidity and stiffness. The stiffness of the leg represents its resistance to compression (flexion of the hip, knee, and ankle joints) during landing, and it is considered one of the parameters that reflect the dynamic stability of the leg. The altered dynamic stability of the leg might increase the likelihood of strain on the articular passive structures that are implied in joint stability, which might contribute to the injury of these structures [35]. Farley and colleagues showed in a study that the primary mechanism for leg stiffness adjustment is the adjustment of ankle stiffness [36]. The difference that we found in the left foot stiffness task in our group of volleyball players might possibly be related to the different training and the different abilities between outside hitter and setter roles on landing on one foot. These players perform jumps from different positions on the court, and their techniques for jumping and landing tasks are different. Proper training after an ankle sprain for returning to the volleyball court is the treatment of ankle stiffness that alters the biomechanics of all the lower limbs and, consequently, of the sport-specific skill.

Talking about other results, our initial hypothesis was that sport-specific tasks, which are different from the roles played on the court in volleyball, could influence biomechanical static and dynamic parameters. Regarding the static postural analysis, the employment of the raster-stereographic-system Formetric-4D postural evaluation technique represents a valuable tool for studying and for imaging acquisition of the entire spine. This device presents a good degree of validity in comparison to exams using X-rays [18,19]. Despite mild differences between the four groups, no statistically significant differences were recognized in the alteration of back alignments during static evaluation using a rastersterographic-system Formetric-4D between groups of volleyball players, separated by role. This might be probably related to the small sample size. The anteroposterior curvatures of the spine in adolescents who practice team sports have been analyzed in the literature, highlighting that different sports might influence the shape of the curvatures due to the tasks and the training performed [37].

Balance has been studied for years as one of the factors that can influence the risk of injury, and several studies have examined balance and proprioceptive training as preventive strategies for specific re-injury in sports [38]. In our study, balance has been investigated through tasks performed with an IMU. The use of IMUs is nowadays common, mostly in élite players, as these units give access to data on kinematic, spatial, and physiological parameters, which can be used to improve physical performance and sport-specific skills [39]. Several protocols have been studied in different environmental conditions to assess their validity for postural control assessment in sportive events [40]. Video assessment has also been suggested in the literature [41]. Differences in static postural control between trained and non-trained people have been suggested in the literature [42]. In particular, postural sway is different between athletes and non-athletes [20]. Sports specializations are possibly related to coordinative differences, which might influence the risk of injury [43]. As sport-specific skills might be different between the roles played in volleyball, we hypothesized that single-leg balance could be different between players, mostly in players who mainly perform jumps and landing on one foot (which is more frequent in the attacking roles) and those who perform jumps on two feet (which is more frequent in defense roles), but we could not be able to highlight differences between groups.

Finally, we investigated how another dynamic task, the CMJ test, could lead to different predispositions for injuries between roles. In a recent study, Miranda-Oliveira and coworkers stated that IMU could serve as a bridge to identify the contraction phase and jump height phase with high accuracy, obtaining a signal similar to that of a force plate, helping coaches and athletes with training monitor and control during their activities [44]. We could not find any statistically significant differences between groups in the CMJ test, in line with another recent study made by Setuain and colleagues [45]. The relationship between take-off and landing phase dynamics and chronic injuries has also been explored in the literature, but there is no evidence in terms of dissimilarities between roles in volleyball [43,46]. In a recent study, Panoutsakopoulos and Bassa evaluated the relationship among ankle flexibility, knee extensor torque, and performance in countermovement jump (CMJ). The authors conclude that a more flexible ankle joint and a higher isokinetic knee extensor torque result in higher CMJ performance [47]. Therefore, training in ankle flexibility should be emphasized, and specific screening should be included during the preseason for youth female volleyball players. Lastly, recent scientific evidence showed that the COVID-19 pandemic might have influenced the prevalence of injuries in sports players [48,49].

Taken together, this study might be considered innovative in its study methodology, considering that, to the best of our knowledge, there is no evidence of postural assessment considering the different predispositions to injury according to the role. On the other hand, this study is not free from limitations. First of all, the small sample size could affect the results obtained in this study. In particular, we highlight that all the participants were right-handed, and this might influence the biomechanics of the sport-specific skills analyzed. Moreover, all the included participants played for teams of the minor league: despite the high number of training sessions per week, different training methods might hamper the analyses of the variables included. Lastly, the group of volleyball players assessed in this study was gender heterogeneous, and this could have a great impact on results. Thus, different training levels might influence the performance of role-specific sports skills. Future studies should aim for a larger, more diverse sample, considering the differences existing in roles and in major versus minor leagues to enhance the generalizability of the findings and to achieve a more in-depth understanding of injury mechanisms and methods for preventing them.

## 5. Conclusions

The purpose of this study was to highlight differences between volleyball roles and sport-specific skills, which could influence the risk of injuries among volleyball players. Implementation of objective evaluation methods of postural analysis, such as raster-stereography, and technological sensors to evaluate both static and dynamic sport-specific skills, such as IMU, are useful in detecting possible alterations of kinematic and relative-kinetic features in sports gestures, such as jumping, or spiking or blocking in volleyball. Moreover, coaches and trainers could be informed, using these highly technological instruments, about intrinsic characteristics and alterations of muscle and tendons of their volleyball players, such as stiffness in the lower limb, preventing injuries and adapting their training according to these data. Besides this, based on our results, it is desirable to carry out further studies that support the use of raster-stereography and IMUs in the evaluation of preventing injuries in volleyball players.

## Figures and Tables

**Table 1:** Demographic characteristics of the included players. Sd = standard deviation.

	Outside Hitter	Setter	Middle Blocker	Opposite Hitter
Number of players (% on the total)	5 (31.25%)	3 (18.75%)	3 (18.75%)	5 (31.25%)
Sex, M/F (%F per group)	¼ (80%)	3 (100%)	½ (66.66%)	2/3 (60%)
Mean Age (sd)	22.4 (±1.48)	20.3 (±2.30)	21 (±1.73)	20.8 (±1.30)
Mean Weight (sd)	64.2 Kg (±10.01)	60 Kg (±5)	62.3 Kg (±3.51)	67.8 Kg (±12.61)
Mean Height (sd)	172.2 cm (±9.36)	175.6 cm (±5.13)	173.3 cm (±3.05)	171.2 cm (±9.20)
Previous Upper Limb Injuries, Yes/No (% Yes)	¼ (20%)	0/3 (0%)	0/3 (0%)	¼ (20%)
Previous Lower Limb Injuries, Yes/No (% Yes)	2/3 (40%)	½ (33.3%)	0/3 (0%)	2/3 (40%)

**Table 2:** Results obtained by Formetric 4D analysis. VP = prominent vertebrae (C7 spinous process); DM = midpoint of the segment joining DR and DL; DR = right lumbar dimple; DL = left lumbar dimple; ICT = cervical-thoracic inversion; ITL = thoracolumbar inversion; ILS = lumbosacral inversion; KA = kyphosis’ apex; LA = lordosis’ apex; rms = root mean square.

	Outside Hitter	Setter	Middle Blocker	Opposite Hitter	*p*
Trunk length VP-DM Median (IQ range)	440.3 (415.5, 468.6)	485.5 (465.6, 492.3)	446.2 (444.7, 448.7)	454.0 (432.3, 465.9)	0.594
Anteroposterior flexion of the spine VP-DM	31.83 (5.21, 40.84)	23.00 (17.59, 35.44)	24.80 (23.98, 52.21)	29.00 (24.63, 57.78)	0.627
Lateral flexion of the spine VP-DM Median (IQ range)	−7.5 (−18.0, −3.0)	−7.5 (−9.75, −6.25)	−8.0 (−8.50, 0.21)	1.50 (−3.00, 3.00)	0.325
Degrees of pelvic inclination DL-DR Median (IQ range)	−1.68 (−1.79, 4.14)	0.00 (−1.50, 2.00)	3.00 (3.00, 3.185)	1.59 (0.00, 6.00)	0.817
Millimeters of pelvic inclination DL-DR Median (IQ range)	−3.00 (−3.00, 7.50)	27.85 (18.43, 27.97)	16.51 (11.26, 17.66)	3.00 (0.00, 8.55)	0.348
Cervical arrow Median (IQ range)	66.28 (59.23, 78.33)	82.00 (76.30, 84.70	79.78 (72.12, 80.89)	76.70 (55.0, 98.59)	0.603
Lumbar arrow Median (IQ range)	38.09 (28.02, 42.98)	47.00 (43.84, 48.08)	38.46 (30.73, 39.48)	32.54 (27.00, 35.87)	0.301
Degrees of kyphotic angle ICT-ITL Median (IQ range)	50.98 (46.87, 52.90)	51.00 (49.46, 53.81)	51.30 (50.27, 54.05)	48.28 (44.33, 49.59)	0.914
Degrees of lordotic angle ITL-ILS Median (IQ range)	44.77 (39.18, 44.84)	46.74 (44.37, 47.56)	40.10 (40.05, 42.73)	47.00 (34.12, 47.06)	0.835
Degrees of pelvic tilt Median (IQ range)	24.07 (23.31, 26.85)	30.87 (22.93, 31.65)	22.01 (19.23, 22.52)	28.52 (15.70, 30.91)	0.806
Degrees of superficial rotation of vertebral bodies_rms Median (IQ range)	3.31 (3.15, 4.90)	2.93 (2.46, 4.24)	1.99 (1.89, 2.51)	3.85 (3.52, 0.31)	0.291
Degrees of superficial rotation of vertebral bodies _amplitude Median (IQ range)	8.81 (6.68, 10.95)	9.46 (7.73, 10.78)	7.15 (6.31, 8.02)	10.50 (9.15, 10.92)	0.48
Lateral deviation VP-DM_rms Median (IQ range)	4.07 (3.24, 5.36)	4.42 (3.71, 6.09)	4.79 (4.43, 5.36)	3.00 (2.79, 6.12)	0.843
Lateral deviation VP-DM_amplitude Median (IQ range)	9.38 (8.99, 16.81)	7.96 (7.48, 14.68)	11.02 (9.81, 11.49)	9.88 (9.00, 12.24)	0.895

**Table 3:** Data on single-leg balance obtained by the inertial sensor. AP = Antero-Posterior; IQ = Interquartile; COM = Center of Mass.

	Outside Hitter	Setter	Middle Blocker	Opposite Hitter	*p*
Antero-Posterior Oscillation of COM on the right foot Median (IQ range)	61.0 (43.0, 91.0)	46 (44, 48.5)	297 (167.5, 515.0)	54.0 (48.0, 58.0)	0.6461
Antero-Posterior Oscillation of COM on the left foot Median (IQ range)	78 (46.0, 97.0)	73.0 (68.5, 84.0)	104 (65.0, 184.0)	85.0 (43.0, 85.0)	0.9716
Lateral Oscillation of COM on the right foot Median (IQ range)	91.0 (37.0, 92.0)	88.0 (65.0, 97.5)	381 (209.5, 450.0)	45 (29, 56)	0.4693
Lateral Oscillation of COM on the left foot Median (IQ range)	59 (34, 80)	94.0 (84.5, 113.0)	223 (127.0, 237.5)	60.0 (38.0, 87.0)	0.4576
Total length of the trajectory of COM on the right foot Median (IQ range)	1349 (785, 1411)	901 (816, 1160)	2487 (1576, 2830)	1037 (890, 1256)	0.9329
Total length of the trajectory of COM on the left foot Median (IQ range)	1091 (1040, 1405)	1278 (980.5, 1329.5)	1409 (998, 1932)	919 (895, 1298)	0.891
Right ellipse area Median (IQ range)	1168 (1101, 2707)	1256 (1166, 1801)	30761 (15,793, 41,080)	1531 (895, 2187)	0.7263
Left ellipse area Median (IQ range)	3192 (1227, 3218)	2708 (2100, 3066	4825 (2664, 16,489)	2053 (621, 3886)	0.9707

**Table 4:** Results of Counter Movement Jump task analysis. IQ = Interquartile.

	Outside Hitter	Setter	Middle Blocker	Opposite Hitter	*p*
Maximum jumping height Median (IQ range)	30 (29, 33)	28.0 (24.5, 29.5)	25.0 (22.5, 34.5)	34.0 (22.0, 34.0)	0.9122
Maximum jumping power Median (IQ range)	1.52 (1.31, 1.67)	1.07 (0.95, 1.14)	1.18 (0.92, 1.45)	1.30 (1.25, 1.60)	0.287
Velocity peak Median (IQ range)	2.50 (2.41, 2.74)	2.30 (2.23, 2.59)	2.16 (1.86, 2.69)	3.10 (2.51, 3.18)	0.468
Total Power Median (IQ range)	1775 (1767, 2216)	1752 (1577, 1755)	1605 (1529, 1850)	2463 (1706, 4094)	0.3277
Stiffness Median (IQ range)	1074.5 (750.2, 1481.5	2116 (2065, 2129)	1158.3 (987.3, 1223.2)	12,589.4 (3169.2, 25,775)	0.1617

**Table 5:** Data on the Stiffness test obtained from the Inertial Motion Unit. IQ = Interquartile; * = statistically significant.

	Outside Hitter	Setter	Middle Blocker	Opposite Hitter	*p*
Right velocity peak Median (IQ range)	1.33 (1.20, 1.48)	1.50 (1.31, 1.58)	1.25 (1.24, 1.47)	1.73 (1.59, 2.31)	0.1718
Left velocity peak Median (IQ range)	1.44 (1.26, 1.50)	1.75 (1.35, 1.76)	1.33 (1.23, 1.45)	1.60 (1.49, 2.17)	0.172
Right maximum jumping height Median (IQ range)	7 (5, 11)	13.00 (9.00, 13.00)	7.00 (7.00, 9.50)	11 (11, 17)	0.244
Left maximum jumping height Median (IQ range)	8 (5.0, 11.0)	13.00 (8.50, 14.00)	8.0 (7.0, 10.5)	11.0 (9.0, 15.0)	0.403
Right stiffness Median (IQ range)	13616 (13,484, 18,326)	10839 (9465, 11,366)	16703 (14,356, 16,741)	12191 (8794, 15,395)	0.462
Left stiffness Median (IQ range)	18469 (14,511, 18,487)	8717 (8339, 9535)	12326 (11,802, 12,598)	10619 (7402, 14,616)	0.0287 *
Right leg force Median (IQ range)	1.24 (1.12, 1.37)	0.86 (0.82, 1.11)	1.20 (1.05, 1.37)	1.46 (1.41, 1.47)	0.285
Left leg force Median (IQ range)	1.22 (1.20, 1.36)	1.06 (0.87, 1.14)	1.13 (0.81, 1.32)	1.22 (1.10, 1.26)	0.357
Right leg Power Median (IQ range)	1013.7 (741.5, 1154.7)	1070.3 (857.6, 1176.1)	930.4 (856.6, 1069.9)	1460.7 (1204.6, 1481.7)	0.165
Left leg power Median (IQ range)	870.7 (776.0, 1004.5)	963.6 (760.0, 1080.7)	974.6 (848.9, 1110.1)	1239 (1115, 1260)	0.213
Right take-off force Median (IQ range)	2.95 (2.51, 2.99)	2.31 (2.14, 2.56)	2.67 (2.55, 3.01)	2.71 (2.59, 2.83)	0.505
Left take-off force Median (IQ range)	2.79 (2.65, 2.81)	2.34 (2.21, 2.42)	2.86 (2.68, 3.04)	2.55 (2.49, 2.74)	0.182

## Data Availability

Data are contained within the article.
